# The function and regulation of TET2 in innate immunity and inflammation

**DOI:** 10.1007/s13238-020-00796-6

**Published:** 2020-10-21

**Authors:** Boyi Cong, Qian Zhang, Xuetao Cao

**Affiliations:** 1 Laboratory of Immunity and Inflammation, College of Life Sciences, Nankai University, Tianjin, 300071 China; 2 National Key Laboratory of Medical Immunology, Institute of Immunology, Navy Military Medical University, Shanghai, 200433 China; 3 Department of Immunology, Center for Immunotherapy, Institute of Basic Medical Sciences, Chinese Academy of Medical Sciences, Beijing, 100005 China

**Keywords:** TET2, innate immune response, DNA demethylation, inflammatory resolution, inflammatory diseases

## Abstract

TET2, a member of ten-eleven translocation (TET) family as α-ketoglutarate- and Fe^2+^-dependent dioxygenase catalyzing the iterative oxidation of 5-methylcytosine (5mC), has been widely recognized to be an important regulator for normal hematopoiesis especially myelopoiesis. Mutation and dysregulation of TET2 contribute to the development of multiple hematological malignancies. Recent studies reveal that TET2 also plays an important role in innate immune homeostasis by promoting DNA demethylation or independent of its enzymatic activity. Here, we focus on the functions of TET2 in the initiation and resolution of inflammation through epigenetic regulation and signaling network. In addition, we highlight regulation of TET2 at various molecular levels as well as the correlated inflammatory diseases, which will provide the insight to intervene in the pathological process caused by TET2 dysregulation.

## Introduction

Innate immune responses are critical in protecting the host from infection and injury, which are efficiently and timely regulated to maintain the immune homeostasis. Diverse epigenetic mechanisms, referring to dynamic regulation of DNA modifications, histone modifications, chromatin remodeling and non-coding RNAs (ncRNAs), are implicated in precise regulation of innate immune responses through establishing specific gene expression patterns especially at transcriptional and post-transcriptional levels (Zhang and Cao, 2019). As the most canonical epigenetic modification, DNA methylation occurs by transferring the methyl group onto 5-carbon of the cytosine to form 5-methylcytosine (5mC), which plays a vital role in transcriptional silencing and genome stability (Jones, 2012), and is of great importance for mammalian development (Jones, 2012; Smith and Meissner, 2013). Aberrant changes of DNA methylation is associated with various pathological diseases, such as cancer (Klutstein et al., 2016), obesity (Zhang et al., 2017a) and inflammatory autoimmune diseases (Meda et al., 2011).

As the chromosomal translocation partner initailly found in leukemia, TET family members, TET1, TET2 and TET3, have been proved to be a key regulator for DNA demethylation owing to its dioxygenase activity. Specifically, TET enzyme oxidizes the methyl group of 5-methylcytosine (5mC) into 5-hydroxymethylcytosine (5hmC), 5-formylcytosine (5fC) and 5-carboxylcytosine (5caC) (Tahiliani et al., 2009; He et al., 2011; Ito et al., 2011), thereby inducing active and passive DNA demethylation through DNA replication or thymine-DNA glycosylase (TDG) and base excision repair (BER) pathway (Wu and Zhang, 2017). TET protein-mediated dynamic regulation of DNA methylation and its oxidation are largely involved in regulating lymphoid and myeloid differentiation and function (Lio and Rao, 2019). In innate immune cells, especially myeloid cells, the expression of TET2 and TET3 is much higher than TET1, and the expression of TET2 increases, while TET3 decreases after TLR ligands stimulation (Zhang et al., 2015; Xue et al., 2016; Cull et al., 2017), implying that TET2 may act as an activation-induced regulator during innate immune response.

Among the three TET family members, TET2 is identified as an important regulator for normal hematopoiesis especially myelopoiesis (Quivoron et al., 2011; Alvarez-Errico et al., 2015). Dysfunction of TET2 is well-proved to be associated with acute myelocytic leukemia (AML), myelodysplastic syndromes (MDS) and other myeloid disorders (Delhommeau et al., 2009; Langemeijer et al., 2009). Recently, numerous studies suggest that TET2 also plays a crucial role in various inflammatory related diseases by regulating innate signaling network and expression of innate effectors during both onset and resolution of immune responses and inflammation. In this review, we provide an overview of the functions of TET2 in innate immunity. In addition, we summarize the regulation of TET2, from transcription, post-transcription, post-translation to gene-specific targeting aspect, and its involvement in inflammatory diseases, thereby implying TET2 as a potential therapeutic target for intervention in inflammatory diseases.

## TET2 in inflammation initiation

Innate immune responses are initiated during pathogen infection and tissue injury by multiple innate immune cells, mainly including macrophages, dendritic cells (DCs), neutrophils, innate lymphoid cells, mast cells, epithelial cells, and endothelial cells (Medzhitov, 2008). Pathogen- or danger-associated molecular patterns (PAMPs or DAMPs) are recognized by germline-encoded pattern-recognition receptors (PRRs) such as Toll-like receptors (TLRs), RIG-I-like receptors (RLRs) and NOD-like receptors (NLRs), which trigger the intracellular signaling pathways to promote the production of various proinflammatory cytokines and mediators (O’Neill et al., 2013; Cao, 2016).

TET2 is required for the differentiation and proliferation of mast cells in mice (Montagner et al., 2016). Loss of TET2 severely changes the 5mC oxidation and the gene expression pattern, which impairs the differentiation of mast cells and the production of cytokines. And TET2 expression is essential for restraining the proliferation of mast cells, independent of its enzymatic activity. Besides, there is evidence that TET2 is involved in regulating the transduction of innate signaling pathways during inflammation initiation. Epigenetic regulator CXXC finger protein 5 (CXXC5) recruits TET2 to maintain hypomethylation of CpG islands (CGI) in the genome of plasmacytoid dendritic cells (pDCs), a rare subset of DCs that highly produce IFN-α in response to viral infection. Among these CGI-associated genes, transcription of *Irf7* is promoted via TET2-sustained promoter hypomethylation, which is critical for TLR-induced type I IFN production for initiating anti-viral immune response (Ma et al., 2017). Besides DNA, TET2 could also act on RNA and perform oxidation of 5mC on RNA in innate immune cells (Fu et al., 2014). TET2 directly binds to the mRNA 3′-UTR of *Socs3*, a negative regulator of JAK-STAT pathway which is important for cytokine-induced myelopoiesis during pathogen infection, and decreases the 5mC level in this region in an enzymatic activity-dependent manner, therefore promoting the degradation of *Socs3* mRNA through ADAR1 and activating the emergency production of mature innate immune cells during pathogen infection (Shen et al., 2018). Although proved to oxidize the 5-methylcytosine on mRNA, whether TET2 can demethylate 5mC in mRNA is still indeterminate, implying that certain cofactors potentially function in this process.

Furthermore, the roles of TET1 and TET3 in innate immunity are also revealed these years. TET3 recruits HDAC1 to the promoter of *Ifnb1* and negatively regulates type I IFN production independent of DNA demethylation (Xue et al., 2016). TET1 is thought to regulate 5hmC in the promoter regions of pro-inflammatory cytokine genes, thereby contributing to the activation of macrophages (Sun et al., 2019).

## TET2 in inflammation resolution

**Figure 1:**
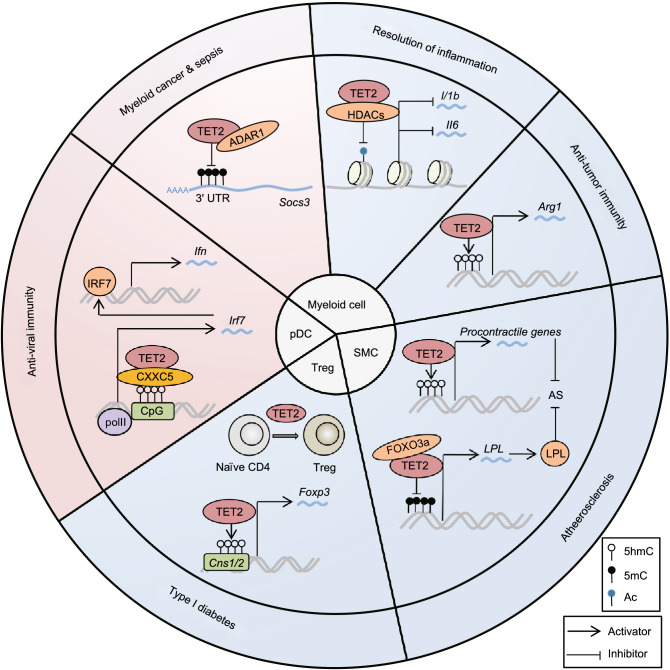
**Negative (blue) or positive (pink) regulation of TET2 in the inflammatory immune responses and related diseases**. During the onset of innate immune response, CXXC5 recruits TET2 to maintain low 5mC level of CpG islands of *Irf7* gene, which promotes IRF7 expression to activate type I IFN production for initiating anti-viral immune response. Besides, TET2 binds to the mRNA 3′-UTR of *Socs3* and inhibits its 5mC level, which promotes the degradation of *Socs3* mRNA through ADAR1 and thereby facilitates infection-induced myelopoiesis. As to the resolution phase of inflammation, TET2, whose expression is promoted via IL-1R-MyD88 pathway, downregulates the expression of inflammatory cytokines IL-6 and IL-1β through recruiting HDACs for histone deacetylation in innate myeloid cells and macrophages respectively. Loss of TET2 in tumor-associated macrophages results in increased expression of inflammatory cytokines as well as decreased expression of ARG1. In addition, TET2 regulates the differentiation of regulatory T cells (Treg) and smooth muscle cells (SMC), thus acting as the repressor of type I diabetes and atherosclerosis

The resolution of inflammation refers to an intricate process in which inflammatory agents are eliminated, inflammatory mediators are catabolized or suppressed, and immune cells including leukocytes are cleared from the inflamed tissue (Buckley et al., 2013). The prompt and effective inflammation resolution is critical for host to maintain the homeostasis and prevent the inflammatory disorders. In recent years, it is increasingly indicated that TET2-involved epigenetic regulation is a key factor for the resolution of inflammation. In an enzymatic activity-independent manner, TET2 binds and recruits HDAC1/2 to facilitate histone deacetylation and suppresses IL-6 and IL-1β expression during the resolution stage of inflammation in innate myeloid cells and macrophages respectively (Zhang et al., 2015; Cull et al., 2017). Loss of TET2 results in upregulation of IL-1β expression and unexpected IL-1β cleavage mediated by NLRP3 inflammasome (Fuster et al., 2017), which confirms the role of TET2 in promoting inflammation resolution. Moreover, tumor-associated macrophages with absent TET2 expression exhibit an immune-active phenotype, including increased expression of inflammatory cytokines and decreased expression of ARG1, which promotes the anti-tumor T cell response (Pan et al., 2017), although another study showed that mutations of DNMT3A and TET2 are most likely associated with the increased expression of ARG1 in bone marrow myelomonocytic cells in MDS/CMML patients (Cull et al., 2018). In summary, these studies indicate that TET2 plays an important role for inflammation resolution by regulating the function of innate immune cells (Fig. 1).

## Transcriptional and post-transcriptional regulation of TET2

Previous studies have identified many transcriptional regulators of TET2 expression. For example, the transcription factor CCAAT/enhancer binding protein alpha (C/EBPα) induces TET2 expression during pre-B cell to macrophage trans-differentiation through binding to the upstream of *TET2* gene (Kallin et al., 2012). Furthermore, the sulfhydrated nuclear transcriptional factor Y subunit beta (NFYB) binds to *TET2* promoter and thereby promotes *TET2* gene transcription (Yang et al., 2015).

**Figure 2:**
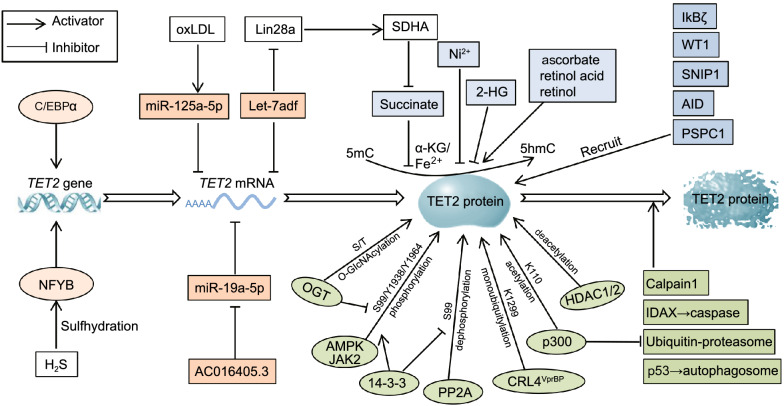
**Regulation of TET2 gene expression and protein function**. Transcription factors, such as C/EBPα and NFYB, promote TET2 expression at mRNA levels, while certain miRNAs and lncRNAs post-transcriptionally regulate TET2 expression. Post-translational modifications (PTMs), including O-GlcNAcylation, phosphorylation, ubiquitylation and acetylation at specific residues respectively, are also involved in regulating TET2 expression. The degradation of TET2 is mediated by four different pathways, namely calpain1, caspase, ubiquitin-proteasome and autophagosome pathway. For some small molecule metabolites, succinate, 2-HG and Ni^2+^ can inhibit TET2 protein activity, while ascorbate, retinol acid and retinol enhance TET2 activity or expression. Transcription factors including IкBζ, WT1 and SNIP1, as well as proteins AID and PSPC1, are able to recruit TET2 to regulate certain gene expression

Expression of TET2 can also be regulated by microRNAs (miRNAs) and long noncoding RNAs (lncRNAs) post-transcriptionally. For instance, miR-125a-5p targets the 3′-UTR of *TET2* mRNA to inhibit TET2 expression during immune responses, thereby activating NLRP3 inflammasome and promoting the expression of proinflammatory cytokine IL-1β and IL-18 (Zhaolin et al., 2019). Let-7a-1/let-7d/let-7f-1 (Let-7adf) microRNA cluster represses TET2 expression or activity through either directly targeting *TET2* mRNA or promoting succinate accumulation by regulating the Lin28a/SDHA axis in LPS-activated macrophages, leading to enhanced production of IL-6 (Jiang et al., 2019) (Fig. 2). Moreover, lncRNA AC016405.3 restrains proliferation and metastasis of glioblastoma multiforme cells through sponging of miR-19a-5p and the subsequent upregulation of TET2 expression (Ren and Xu, 2019).

## Post-translational regulation of TET2

### Degradation

It has been reported that TET2 is degraded via four different pathways. CXXC domain-containing protein IDAX (also known as CXXC4) can promote caspase activation probably via regulating gene transcription. As a TET2 binding partner, IDAX promotes degradation of TET2 protein (Ko et al., 2013). A family member of calcium-dependent proteases, calpain1, mediates TET2 degradation, which is likely to affect global 5hmC level and expression of certain lineage-specific genes in mESCs (Wang and Zhang, 2014). The degradation of TET2 can also be mediated by the ubiquitin-proteasome pathway that poly-ubiquitination of the C-terminal conserved DSBH domain increases the instability of TET2 (Lv et al., 2018). Recently, it was proposed that p53 facilitates the autophagic degradation of TET2 by promoting the shuttling of nuclear TET2 toward the cytoplasmic autophagosome (Zhang et al., 2019a).

### Monoubiquitylation and acetylation

TET2 binds to VprBP and is monoubiquitylated by the CRL4^VprBP^ E3 ubiquitin ligase on a highly conserved lysine residue, which facilitates TET2 binding to chromatin, although CRL4^VprBP^ also can destabilize TET2 through lysine poly-ubiquitination (Nakagawa et al., 2015; Lv et al., 2018). TET2 is acetylated at lysine 110 by p300, which stabilizes TET2 protein through the inhibition of TET2 ubiquitination. Consequently, the recruitment of TET2 to chromatin during oxidative stress is promoted to prevent abnormal DNA methylation (Zhang et al., 2017b).

### O-glcNAcylation and phosphorylation

O-linked GlcNAc transferase (OGT) catalyzes TET2 O-GlcNAcylation and thereby reduces TET2 phosphorylation at the N terminus and low-complexity insert region (Bauer et al., 2015). AMPK phosphorylates TET2 and enhances TET2 stability (Zhang et al., 2019b). Increased glucose levels hinder AMPK-mediated human TET2 phosphorylation at serine 99, resulting in the destabilization of TET2 followed by dysregulation of both 5hmC level and the tumor suppressive effect of TET2 *in vitro* and *in vivo* (Wu et al., 2018). 14-3-3 protein can bind TET2 to maintain AMPK-mediated serine 99 phosphorylation via protecting TET2 from protein phosphatase 2A (PP2A)-mediated dephosphorylation (Kundu et al., 2020). Besides, JAK2 can also phosphorylate TET2 at tyrosine 1939 and 1964 which increases TET2 activity and enhances binding of transcription factor KLF1 with TET2 upon hematopoietic cytokine erythropoietin (EPO) stimuli (Jeong et al., 2019).

### Metabolic factors

TET2-mediated oxidation of 5mC can also be regulated during metabolic reprogramming (Yang et al., 2014). TET proteins belong to 2-oxoglutarate oxygenases, employing Fe^2+^ as metal cofactor and α-KG as co-substrate (Tahiliani et al., 2009; Loenarz and Schofield, 2011). α-KG-dependent TET2 enzymatic activity is suppressed due to the accumulation of metabolites that share structural similarity with α-KG, such as succinate which inhibits the oxidation of 5mC to 5hmC (Killian et al., 2013) and 2-HG (Xu et al., 2011). Also, natural Ni^2+^ ion can displace Fe^2+^, the cofactor of TET2, thus inhibiting TET2 enzymatic activity (Yin et al., 2017). Besides, retinol acid, retinol (vitamin A) and ascorbate (vitamin C) are proved to regulate TET2 activity or expression. Ascorbate is likely to act as a cofactor of TET2 (Minor et al., 2013), which enhances TET2 enzymatic activity and alter DNA methylation so as to regulate the expression of germline genes in ES cells (Blaschke et al., 2013). Another study proposed that ascorbate enhances the catalytic activity of TET2 and the production of 5hmC by reducing Fe^3+^ to Fe^2+^ that participates in the catalytic center of TET2 (Hore et al., 2016). Without affecting TET2 protein stability or catalytic efficiency, retinol acid or retinol (vitamin A) activates TET2 expression probably through affecting *TET2* transcription, and thereby enhances 5hmC production in naive embryonic stem cells (Hore et al., 2016).

## Recruitment of TET2

As to the structural characteristic of TET proteins, both full-length TET1 and TET3 contain a CXXC domain at the amino terminus that helps them bind DNA, whereas TET2 does not (Pastor et al., 2013). Various proteins are reported to be involved in the recruitment of TET2 to target chromatin in different biological processes. Transcription factors, including IкBζ (Zhang et al., 2015), WT1 (Wang et al., 2015), and SNIP1 (Chen et al., 2018), mediate the recruitment of TET2 to specific gene loci to regulate gene transcription. IDAX preferentially binds to CpG-rich regions containing unmodified cytosines in gene promoter regions and CpG islands through its CXXC domain in a DNA sequence-independent manner. IDAX interacts with TET2 catalytic domain and recruits TET2 to DNA (Ko et al., 2013). Besides, activation-induced cytidine deaminase (AID) can recruit TET2 to *FANCA* promoter to induce demethylation for oncogene activation in diffuse large B cell lymphoma (Jiao et al., 2019).

Furthermore, TET2 can potentially be recruited by some RNA-binding proteins, which may lead to a broader regulatory role of TET2, on regulating RNA splicing, stability, localization and degradation. RNA-binding protein Paraspeckle component 1 (PSPC1) recruits TET2 to transcriptionally active loci in an RNA-dependent manner, leading to the RNA 5hmC modification and the destabilization of *MERVL* RNAs through unidentified mechanism. PSPC1-TET2 complex can also recruit HDAC1/2 for transcriptional silencing of *MERVL* transcripts via histone deacetylation (Guallar et al., 2018). A previous study identified a nine-residue peptide adjacent to the catalytic domain of TET2 as the most likely RNA binding site (He et al., 2016), suggesting that TET2 itself acts as a RNA-binding protein and is involved in the post-transcriptional regulation of gene expression. Further study also proved the RNA-binding capacity of TET2 through enhanced crosslinking and immunoprecipitation followed by high-throughput sequencing (eCLIP-seq) (Shen et al., 2018).

## TET2 and inflammatory diseases

### Atherosclerosis

As described above, inhibition of TET2 upregulates the production of mature proinflammatory cytokines including IL-1β and IL-18, which activates the inflammation response and subsequently contributes to and accelerates atherosclerosis (Fuster et al., 2017; Zhaolin et al., 2019). TET2 has been identified as a key epigenetic regulator of SMC differentiation and phenotype switch of vascular SMC to a pro-proliferation and migration phenotype (Liu et al., 2013; Li et al., 2020). TET2 facilitates the expression of key procontractile genes in SMCs inducing the contractile phenotype of SMCs, thus attenuates vascular injury in human atherosclerotic disease (Liu et al., 2013). Additionally, coiled-coil domain-containing 80 (CCDC80) inhibits the phosphorylation of ERK1/2 and decreases the expression of TET2, which upregulates the methylation level of lipoprotein lipase (LPL) promoter region, and impairs the interaction of TET2 with the transcription factor FOXO3a, causing a reduction of LPL expression and finally the acceleration of atherosclerosis (Gong et al., 2019).

### Type I diabetes

Type I diabetes (T1D) is caused by autoimmune damage of pancreatic β cells, and epigenetic regulation has been proposed to involve in the progression of T1D (MacFarlane et al., 2009). As mentioned above, a study revealed a regulatory pathway that links glucose and AMPK to TET2 and 5hmC (Wu et al., 2018). Expression of IFN-α in pancreatic islets during acute or chronic infections, post-transcriptionally increases expression of TET2 through targeting miR-26a for degradation, which in turn increases global 5hmC level of pancreatic β cells, resulting in the initiation of islet autoimmunity in T1D (Stefan-Lifshitz et al., 2019). Moreover, TET2 mediates the demethylation of two intronic enhancers *CNS1* and *CNS2* and stabilizes FOXP3 expression in Treg (Yang et al., 2015; Yue et al., 2016, 2019). The decreased expression of TET2 was demonstrated in Tregs from both mice T1D model and human T1D patients, which impairs Treg stability and function and results in islet autoimmune response (Scherm et al., 2019). In addition, TET2 also plays a role in the pathogenesis of diabetic nephropathy (DN) by activating TGF-β1 expression through demethylation of CpG islands in the TGF-β1 regulatory region (Yang et al., 2018).

### Autoimmune diseases

A decreased state of global DNA methylation is verified in patients with rheumatoid arthritis (RA). Correspondingly, the expression of TET2 is increased in the monocytes and T cells of RA patients, which supports that DNA hypomethylation and TET2 enzymes are associated with RA (de Andres et al., 2015). TET2 expression is also a potential prognostic and predictive biomarker in cytogenetically normal acute myeloid leukemia (Zhang et al., 2018). HIV-1 Vpr protein promotes degradation of TET2, thereby attenuating its binding to *Il-6* promoter region, which causes excessive IL-6 expression during resolution phase of inflammation (Lv et al., 2018). A recent study found that loss of TET2 and TET3 upregulates CD86 expression through relieving the gene-specific transcription repression by HDAC1/2, thus leads to hyperactivation of B cells and T cells in mice (Tanaka et al., 2020). Consequently, these mice are prone to develop systemic autoimmunity, which implies the function of TET2 in lupus-like diseases.

## Conclusions and perspectives

Epigenetic regulation plays an important role in modulating immune responses against infection or injury. Numerous studies have well-indicated the significance of TET2 proteins and 5hmC in epigenetic regulation, hematopoietic stem cell development and myelopoiesis. The studies discussed in this review demonstrate the emerging roles of TET2, depending on its enzymatic activity or acting as a scaffold protein, in the homeostatic regulation of immune responses and pathogenesis of inflammatory diseases. We also discuss how TET2 is regulated and recruited, which provides a promising thought to potentially alter the process of immune responses. Despite so many discovered functions of TET2 in immune responses and inflammation, there are still many questions that need to be answered. Are there any other specific mechanisms of TET2 involved in the onset and resolution of inflammation? Can TET2 mutations be detected in other inflammatory diseases? Over the past decades, remarkable progress has been made in exploring the function of TET2 in regulating DNA methylation/demethylation by mediating the oxidation of 5mC. However, can oxidative modifications catalyzed by TET2 directly regulate gene expression as an independent type of epigenetic modifications, not just as the intermediate step of active DNA demethylation process? Besides, because of the distinct structure of TET2 compared to TET1 and TET3, the mechanism of RNA-dependent chromatin targeting of TET2 is still to be further studied. Is there a general regulation mechanism rather than a specific one of how TET2 is recruited to chromatin? Thus, more partner proteins of TET2 need to be identified. On the other hand, the mechanisms of how TET2 regulates gene expression by directly binding RNA are researchable. Combining with multiple epigenomic methods, many novel technologies have been developed and applied to screen the genomic distribution of TET2 catalyzing oxidation modifications and investigate more probable functions of TET2. Most importantly, further studies are required to illuminate the potential therapeutic role of targeting TET2 for the modulation of immune responses and the treatment of relevant inflammatory diseases.

## References

[CR1] Alvarez-Errico D, Vento-Tormo R, Sieweke M, Ballestar E (2015). Epigenetic control of myeloid cell differentiation, identity and function. Nat Rev Immunol.

[CR2] Bauer C, Gobel K, Nagaraj N, Colantuoni C, Wang M, Muller U, Kremmer E, Rottach A, Leonhardt H (2015). Phosphorylation of TET proteins is regulated via O-GlcNAcylation by the O-linked N-acetylglucosamine transferase (OGT). J Biol Chem.

[CR3] Blaschke K, Ebata KT, Karimi MM, Zepeda-Martinez JA, Goyal P, Mahapatra S, Tam A, Laird DJ, Hirst M, Rao A (2013). Vitamin C induces Tet-dependent DNA demethylation and a blastocyst-like state in ES cells. Nature.

[CR4] Buckley CD, Gilroy DW, Serhan CN, Stockinger B, Tak PP (2013). The resolution of inflammation. Nat Rev Immunol.

[CR5] Cao X (2016). Self-regulation and cross-regulation of pattern-recognition receptor signalling in health and disease. Nat Rev Immunol.

[CR6] Chen LL, Lin HP, Zhou WJ, He CX, Zhang ZY, Cheng ZL, Song JB, Liu P, Chen XY, Xia YK (2018). SNIP1 recruits TET2 to regulate c-MYC target genes and cellular DNA damage response. Cell Rep.

[CR7] Cull AH, Mahendru D, Snetsinger B, Good D, Tyryshkin K, Chesney A, Ghorab Z, Reis M, Buckstein R, Wells RA (2018). Overexpression of Arginase 1 is linked to DNMT3A and TET2 mutations in lower-grade myelodysplastic syndromes and chronic myelomonocytic leukemia. Leuk Res.

[CR8] Cull AH, Snetsinger B, Buckstein R, Wells RA, Rauh MJ (2017). TET2 restrains inflammatory gene expression in macrophages. Exp Hematol.

[CR9] de Andres MC, Perez-Pampin E, Calaza M, Santaclara FJ, Ortea I, Gomez-Reino JJ, Gonzalez A (2015). Assessment of global DNA methylation in peripheral blood cell subpopulations of early rheumatoid arthritis before and after methotrexate. Arthritis Res Ther.

[CR10] Delhommeau F, Dupont S, Della Valle V, James C, Trannoy S, Masse A, Kosmider O, Le Couedic JP, Robert F, Alberdi A (2009). Mutation in TET2 in myeloid cancers. N Engl J Med.

[CR11] Fu L, Guerrero CR, Zhong N, Amato NJ, Liu Y, Liu S, Cai Q, Ji D, Jin SG, Niedernhofer LJ (2014). Tet-mediated formation of 5-hydroxymethylcytosine in RNA. J Am Chem Soc.

[CR12] Fuster JJ, MacLauchlan S, Zuriaga MA, Polackal MN, Ostriker AC, Chakraborty R, Wu CL, Sano S, Muralidharan S, Rius C (2017). Clonal hematopoiesis associated with TET2 deficiency accelerates atherosclerosis development in mice. Science.

[CR13] Gong D, Zhang Q, Chen LY, Yu XH, Wang G, Zou J, Zheng XL, Zhang DW, Yin WD, Tang CK (2019). Coiled-coil domain-containing 80 accelerates atherosclerosis development through decreasing lipoprotein lipase expression via ERK1/2 phosphorylation and TET2 expression. Eur J Pharmacol.

[CR14] Guallar D, Bi X, Pardavila JA, Huang X, Saenz C, Shi X, Zhou H, Faiola F, Ding J, Haruehanroengra P (2018). RNA-dependent chromatin targeting of TET2 for endogenous retrovirus control in pluripotent stem cells. Nat Genet.

[CR15] He C, Sidoli S, Warneford-Thomson R, Tatomer DC, Wilusz JE, Garcia BA, Bonasio R (2016). High-resolution mapping of RNA-binding regions in the nuclear proteome of embryonic stem cells. Mol Cell.

[CR16] He YF, Li BZ, Li Z, Liu P, Wang Y, Tang Q, Ding J, Jia Y, Chen Z, Li L (2011). Tet-mediated formation of 5-carboxylcytosine and its excision by TDG in mammalian DNA. Science.

[CR17] Hore TA, von Meyenn F, Ravichandran M, Bachman M, Ficz G, Oxley D, Santos F, Balasubramanian S, Jurkowski TP, Reik W (2016). Retinol and ascorbate drive erasure of epigenetic memory and enhance reprogramming to naive pluripotency by complementary mechanisms. Proc Natl Acad Sci USA.

[CR18] Ito S, Shen L, Dai Q, Wu SC, Collins LB, Swenberg JA, He C, Zhang Y (2011). Tet proteins can convert 5-methylcytosine to 5-formylcytosine and 5-carboxylcytosine. Science.

[CR19] Jeong JJ, Gu X, Nie J, Sundaravel S, Liu H, Kuo WL, Bhagat TD, Pradhan K, Cao J, Nischal S (2019). Cytokine-regulated phosphorylation and activation of TET2 by JAK2 in hematopoiesis. Cancer Discov.

[CR20] Jiang S, Yan W, Wang SE, Baltimore D (2019). Dual mechanisms of posttranscriptional regulation of TET2 by Let-7 microRNA in macrophages. Proc Natl Acad Sci USA.

[CR21] Jiao J, Jin Y, Zheng M, Zhang H, Yuan M, Lv Z, Odhiambo W, Yu X, Zhang P, Li C (2019). AID and TET2 co-operation modulates FANCA expression by active demethylation in diffuse large B cell lymphoma. Clin Exp Immunol.

[CR22] Jones PA (2012). Functions of DNA methylation: islands, start sites, gene bodies and beyond. Nat Rev Genet.

[CR23] Kallin EM, Rodriguez-Ubreva J, Christensen J, Cimmino L, Aifantis I, Helin K, Ballestar E, Graf T (2012). TET2 facilitates the derepression of myeloid target genes during CEBPalpha-induced transdifferentiation of pre-B cells. Mol Cell.

[CR24] Killian JK, Kim SY, Miettinen M, Smith C, Merino M, Tsokos M, Quezado M, Smith WI, Jahromi MS, Xekouki P (2013). Succinate dehydrogenase mutation underlies global epigenomic divergence in gastrointestinal stromal tumor. Cancer Discov.

[CR25] Klutstein M, Nejman D, Greenfield R, Cedar H (2016). DNA methylation in cancer and aging. Cancer Res.

[CR26] Ko M, An J, Bandukwala HS, Chavez L, Aijo T, Pastor WA, Segal MF, Li H, Koh KP, Lahdesmaki H (2013). Modulation of TET2 expression and 5-methylcytosine oxidation by the CXXC domain protein IDAX. Nature.

[CR27] Kundu A, Shelar S, Ghosh A, Ballestas M, Kirkman R, Nam HY, Brinkley G, Karki S, Mobley JA, Bae S (2020). 14-3-3 proteins protect AMPK-phosphorylated ten-eleven translocation-2 (TET2) from PP2A-mediated dephosphorylation. J Biol Chem..

[CR28] Langemeijer SM, Kuiper RP, Berends M, Knops R, Aslanyan MG, Massop M, Stevens-Linders E, van Hoogen P, van Kessel AG, Raymakers RA (2009). Acquired mutations in TET2 are common in myelodysplastic syndromes. Nat Genet.

[CR29] Li B, Zang G, Zhong W, Chen R, Zhang Y, Yang P, Yan J (2020). Activation of CD137 signaling promotes neointimal formation by attenuating TET2 and transferrring from endothelial cell-derived exosomes to vascular smooth muscle cells. Biomed Pharmacother.

[CR30] Lio CJ, Rao A (2019). TET enzymes and 5hmC in adaptive and innate immune systems. Front Immunol.

[CR31] Liu R, Jin Y, Tang WH, Qin L, Zhang X, Tellides G, Hwa J, Yu J, Martin KA (2013). Ten-eleven translocation-2 (TET2) is a master regulator of smooth muscle cell plasticity. Circulation.

[CR32] Loenarz C, Schofield CJ (2011). Physiological and biochemical aspects of hydroxylations and demethylations catalyzed by human 2-oxoglutarate oxygenases. Trends Biochem Sci.

[CR33] Lv L, Wang Q, Xu Y, Tsao LC, Nakagawa T, Guo H, Su L, Xiong Y (2018). Vpr targets TET2 for degradation by CRL4(VprBP) E3 ligase to sustain IL-6 expression and enhance HIV-1 replication. Mol Cell.

[CR34] Ma S, Wan X, Deng Z, Shi L, Hao C, Zhou Z, Zhou C, Fang Y, Liu J, Yang J (2017). Epigenetic regulator CXXC5 recruits DNA demethylase TET2 to regulate TLR7/9-elicited IFN response in pDCs. J Exp Med.

[CR35] MacFarlane AJ, Strom A, Scott FW (2009). Epigenetics: deciphering how environmental factors may modify autoimmune type 1 diabetes. Mamm Genome.

[CR36] Meda F, Folci M, Baccarelli A, Selmi C (2011). The epigenetics of autoimmunity. Cell Mol Immunol.

[CR37] Medzhitov R (2008). Origin and physiological roles of inflammation. Nature.

[CR38] Minor EA, Court BL, Young JI, Wang G (2013). Ascorbate induces ten-eleven translocation (Tet) methylcytosine dioxygenase-mediated generation of 5-hydroxymethylcytosine. J Biol Chem.

[CR39] Montagner S, Leoni C, Emming S, Della Chiara G, Balestrieri C, Barozzi I, Piccolo V, Togher S, Ko M, Rao A (2016). TET2 regulates mast cell differentiation and proliferation through catalytic and non-catalytic activities. Cell Rep.

[CR40] Nakagawa T, Lv L, Nakagawa M, Yu Y, Yu C, D’Alessio AC, Nakayama K, Fan HY, Chen X, Xiong Y (2015). CRL4(VprBP) E3 ligase promotes monoubiquitylation and chromatin binding of TET dioxygenases. Mol Cell.

[CR41] O’Neill LA, Golenbock D, Bowie AG (2013). The history of Toll-like receptors - redefining innate immunity. Nat Rev Immunol.

[CR42] Pan W, Zhu S, Qu K, Meeth K, Cheng J, He K, Ma H, Liao Y, Wen X, Roden C (2017). The DNA methylcytosine dioxygenase TET2 sustains immunosuppressive function of tumor-infiltrating myeloid cells to promote melanoma progression. Immunity.

[CR43] Pastor WA, Aravind L, Rao A (2013). TETonic shift: biological roles of TET proteins in DNA demethylation and transcription. Nat Rev Mol Cell Biol.

[CR44] Quivoron C, Couronne L, Della Valle V, Lopez CK, Plo I, Wagner-Ballon O, Do Cruzeiro M, Delhommeau F, Arnulf B, Stern MH (2011). TET2 inactivation results in pleiotropic hematopoietic abnormalities in mouse and is a recurrent event during human lymphomagenesis. Cancer Cell.

[CR45] Ren S, Xu Y (2019). AC016405.3, a novel long noncoding RNA, acts as a tumor suppressor through modulation of TET2 by microRNA-19a-5p sponging in glioblastoma. Cancer Sci.

[CR46] Scherm MG, Serr I, Zahm AM, Schug J, Bellusci S, Manfredini R, Salb VK, Gerlach K, Weigmann B, Ziegler AG (2019). miRNA142-3p targets TET2 and impairs Treg differentiation and stability in models of type 1 diabetes. Nat Commun.

[CR47] Shen Q, Zhang Q, Shi Y, Shi Q, Jiang Y, Gu Y, Li Z, Li X, Zhao K, Wang C (2018). TET2 promotes pathogen infection-induced myelopoiesis through mRNA oxidation. Nature.

[CR48] Smith ZD, Meissner A (2013). DNA methylation: roles in mammalian development. Nat Rev Genet.

[CR49] Stefan-Lifshitz M, Karakose E, Cui L, Ettela A, Yi Z, Zhang W, Tomer Y (2019). Epigenetic modulation of beta cells by interferon-alpha via PNPT1/mir-26a/TET2 triggers autoimmune diabetes. JCI Insight.

[CR50] Sun F, Abreu-Rodriguez I, Ye S, Gay S, Distler O, Neidhart M, Karouzakis E (2019). TET1 is an important transcriptional activator of TNFalpha expression in macrophages. PLoS One.

[CR51] Tahiliani M, Koh KP, Shen Y, Pastor WA, Bandukwala H, Brudno Y, Agarwal S, Iyer LM, Liu DR, Aravind L (2009). Conversion of 5-methylcytosine to 5-hydroxymethylcytosine in mammalian DNA by MLL partner TET1. Science.

[CR52] Tanaka S, Ise W, Inoue T, Ito A, Ono C, Shima Y, Sakakibara S, Nakayama M, Fujii K, Miura I (2020). TET2 and Tet3 in B cells are required to repress CD86 and prevent autoimmunity. Nat Immunol..

[CR53] Wang Y, Xiao M, Chen X, Chen L, Xu Y, Lv L, Wang P, Yang H, Ma S, Lin H (2015). WT1 recruits TET2 to regulate its target gene expression and suppress leukemia cell proliferation. Mol Cell.

[CR54] Wang Y, Zhang Y (2014). Regulation of TET protein stability by calpains. Cell Rep.

[CR55] Wu D, Hu D, Chen H, Shi G, Fetahu IS, Wu F, Rabidou K, Fang R, Tan L, Xu S (2018). Glucose-regulated phosphorylation of TET2 by AMPK reveals a pathway linking diabetes to cancer. Nature.

[CR56] Wu X, Zhang Y (2017). TET-mediated active DNA demethylation: mechanism, function and beyond. Nat Rev Genet.

[CR57] Xu W, Yang H, Liu Y, Yang Y, Wang P, Kim SH, Ito S, Yang C, Wang P, Xiao MT (2011). Oncometabolite 2-hydroxyglutarate is a competitive inhibitor of alpha-ketoglutarate-dependent dioxygenases. Cancer Cell.

[CR58] Xue S, Liu C, Sun X, Li W, Zhang C, Zhou X, Lu Y, Xiao J, Li C, Xu X (2016). TET3 Inhibits Type I IFN Production Independent of DNA Demethylation. Cell Rep.

[CR59] Yang H, Lin H, Xu H, Zhang L, Cheng L, Wen B, Shou J, Guan K, Xiong Y, Ye D (2014). TET-catalyzed 5-methylcytosine hydroxylation is dynamically regulated by metabolites. Cell Res.

[CR60] Yang L, Zhang Q, Wu Q, Wei Y, Yu J, Mu J, Zhang J, Zeng W, Feng B (2018). Effect of TET2 on the pathogenesis of diabetic nephropathy through activation of transforming growth factor beta1 expression via DNA demethylation. Life Sci.

[CR61] Yang R, Qu C, Zhou Y, Konkel JE, Shi S, Liu Y, Chen C, Liu S, Liu D, Chen Y (2015). Hydrogen sulfide promotes Tet1- and TET2-mediated Foxp3 demethylation to drive regulatory T cell differentiation and maintain immune homeostasis. Immunity.

[CR62] Yin R, Mo J, Dai J, Wang H (2017). Nickel(II) Inhibits Tet-mediated 5-methylcytosine oxidation by high affinity displacement of the cofactor iron(II). ACS Chem Biol.

[CR63] Yue X, Lio CJ, Samaniego-Castruita D, Li X, Rao A (2019). Loss of TET2 and TET3 in regulatory T cells unleashes effector function. Nat Commun.

[CR64] Yue X, Trifari S, Aijo T, Tsagaratou A, Pastor WA, Zepeda-Martinez JA, Lio CW, Li X, Huang Y, Vijayanand P (2016). Control of Foxp3 stability through modulation of TET activity. J Exp Med.

[CR68] Zhang Q, Zhao K, Shen Q, Han Y, Gu Y, Li X, Zhao D, Liu Y, Wang C, Zhang X (2015). TET2 is required to resolve inflammation by recruiting Hdac2 to specifically repress IL-6. Nature.

[CR66] Zhang P, Chu T, Dedousis N, Mantell BS, Sipula I, Li L, Bunce KD, Shaw PA, Katz LS, Zhu J (2017). DNA methylation alters transcriptional rates of differentially expressed genes and contributes to pathophysiology in mice fed a high fat diet. Mol Metab.

[CR71] Zhang YW, Wang Z, Xie W, Cai Y, Xia L, Easwaran H, Luo J, Yen RC, Li Y, Baylin SB (2017). Acetylation enhances TET2 function in protecting against abnormal DNA methylation during oxidative stress. Mol Cell.

[CR70] Zhang TJ, Zhou JD, Yang DQ, Wang YX, Wen XM, Guo H, Yang L, Lian XY, Lin J, Qian J (2018). TET2 expression is a potential prognostic and predictive biomarker in cytogenetically normal acute myeloid leukemia. J Cell Physiol.

[CR67] Zhang Q, Cao X (2019). Epigenetic regulation of the innate immune response to infection. Nat Rev Immunol..

[CR65] Zhang J, Tan P, Guo L, Gong J, Ma J, Li J, Lee M, Fang S, Jing J, Johnson G (2019). p53-dependent autophagic degradation of TET2 modulates cancer therapeutic resistance. Oncogene.

[CR69] Zhang T, Guan X, Choi UL, Dong Q, Lam MMT, Zeng J, Xiong J, Wang X, Poon TCW, Zhang H (2019). Phosphorylation of TET2 by AMPK is indispensable in myogenic differentiation. Epigenetics Chromatin.

[CR72] Zhaolin Z, Jiaojiao C, Peng W, Yami L, Tingting Z, Jun T, Shiyuan W, Jinyan X, Dangheng W, Zhisheng J (2019). OxLDL induces vascular endothelial cell pyroptosis through miR-125a-5p/TET2 pathway. J Cell Physiol.

